# Comparison of the inhibitory effect of topical cyclosporine A 0.1% and topical anti-VEGF application in an experimental model of corneal neovascularization

**DOI:** 10.5935/0004-2749.20220004

**Published:** 2022

**Authors:** Döndü Melek Ulusoy, Nisa Kahraman, Esra Balcıoğlu, Zeynep Duru

**Affiliations:** 1 Ophtalmology Department, Kayseri Training and Research Hospital, Kayseri, Turkey; 2 Department of Histology and Embryology, Faculty of Medicine, Erciyes University, Kayseri, Turkey

**Keywords:** Corneal neovascularization, Bevacizumab, Cyclosporine A, Rats, Neovascularização da córnea, Bevacizumabe, Cyclosporina A, Ratos

## Abstract

**Purpose:**

The aim of this study was to compare the effects of topical cyclosporine 0.1% and bevacizumab on experimentally induced corneal neovascularization in a rat model.

**Methods:**

A total of 30 adult Sprague-Dawley rats were used in this experimental study. The central cornea of the rats was cauterized chemically. The rats were randomly enrolled into three groups as follows: Group 1 received bevacizumab 1%, Group 2 received cyclosporine 0.1%, and Group 3 received isotonic saline twice a day for 28 days. Slit-lamp examination of all rats was performed at the 3rd and 28th day. The rats were then sacrificed, and the corneas were excised. The number of blood vessels, state of inflammation, and collagen formation were evaluated histopathologically in the corneal sections.

**Results:**

Corneal opacity and edema grades were significantly lower in Group 2 than in Group 3 (p=0.04 and 0.00, respectively). In the histopathological examination, Group 2 demonstrated significantly lesser number of blood vessels than Group 3 (p=0.001). Regarding collagen formation, Group 2 exhibited more regular collagen formation than Groups 1 and 3 (p=0.03). Inflammation grades were significantly lower in Groups 1 and 2 than in Group 3 (p=0.014 and 0.001, respectively).

**Conclusion:**

Topical bevacizumab is effective in inhibiting newly formed corneal neovascularization. The topical cyclosporine 0.1% treatment appears to be more effective than the topical bevacizumab treatment.

## Abstract (pt)

**Objetivo:**

Comparar os efeitos da ciclosporina tópica 0,1% e do bevacizumabe na neovascularização da córnea produzida experimentalmente em um modelo com ratos.

**Métodos:**

Trinta ratos Sprague-Dawley adultos foram usados neste estudo experimental. A córnea central dos ratos foi cauterizada quimicamente. Os ratos foram distribuídos aleatoriamente em três grupos. O grupo 1 recebeu bevacizumabe a 1%, o grupo 2 recebeu ciclosporina tópica a 0,1% e o grupo 3 recebeu solução salina isotônica duas vezes ao dia durante 28 dias. O exame de lâmpada de fenda de todos os ratos foi realizado no terceiro e no vigésimo oitavo dias. Os ratos foram então sacrificados e as córneas excisadas. Nos cortes da córnea, o número de vasos sanguíneos, o estado de inflamação e a formação de colágeno foram avaliados em uma análise anatomopatológica.

**Resultados:**

No Grupo 2, os graus de opacidade e de edema da córnea foram significativamente menores que no Grupo 3 (p=0,04 e 0,00, respectivamente). No exame histopatológico, o Grupo 2 apresentou um número significativamente menor de vasos sanguíneos do que o Grupo 3 (p=0,001). Em relação à avaliação da formação de colágeno, esta mostrou-se mais regular no Grupo 2 que no Grupo 1 e no Grupo 3 (p=0,03). Os graus de inflamação foram significativamente menores no Grupo 1 e no Grupo 2 em comparação com o Grupo 3 (p=0,014 e 0,001, respectivamente).

**Conclusão:**

O bevacizumabe tópico é eficaz na inibição da neovascularização da córnea recém-formada. O tratamento tópico com ciclosporina a 0,1% parece ser mais eficaz em comparação ao tratamento tópico com bevacizumabe.

## INTRODUCTION

The cornea breaks the light coming to the eye and serves as a mechanical barrier. It is normally a nonvascularized transparent tissue. Avascularity is required for the maintenance of corneal transparency^(1)^. Chemical burns of the cornea result in superficial and deep neovascularization that can cause deterioration of the transparency due to scar formation and lipid deposition^(2,3)^. Corneal neovascularization (CNV) occurs when the balance between angiogenic and antiangiogenic factors is impaired due to the upregulation of angiogenic factors and/or the downregulation of antiangiogenic factors^(3)^. Although the clinical relevance of CNV has long been recognized, its treatment remains challenging and is associated with varying degrees of success^(4-6)^. Currently available options for CNV treatment include pharmacological approaches such as corticosteroids and nonsteroidal anti-inflammatory agents, both of which may be associated with side effects. Other treatment options include metalloproteinase inhibitors and monoclonal antibodies targeting the vascular endothelial growth factor (VEGF), among others^(4,5)^. Anti-VEGF antibodies, such as bevacizumab, have several disadvantages despite the good results reported by different authors^(6)^. Moreover, bevacizumab is used off-label for this purpose. Due to these reasons, there is a need for alternative, effective, and safe treatment approaches for CNV.

Cyclosporine A (CsA) is a T-cell-specific immunosuppressive drug used to control the rejection of organ transplants and to treat various autoimmune and inflammatory conditions^(7)^. In the treatment of several ocular inflammatory and immune diseases, CsA has generally been used in clinical practice in the form of eye drops or ointment at concentrations between 0.05% and 2.5%. However, the therapeutic potential of topical CsA 0.1% in treating CNV has not been explored till date.

Therefore, considering the lack of a specific and approved drug for the treatment of CNV, we conducted this study to compare the effects of topical cyclosporine and bevacizumab on experimentally induced CNV in a rat model.

## METHODS

This study was conducted at the Kayseri Training and Research Hospital, with the necessary approval being obtained from the Animal Ethics Committee. A total of 30 male Sprague-Dawley rats weighing 250-300 g and with two healthy eyes were used in this study. The animals were treated and maintained according to the tenets of the Association for Research in Vision and Ophthalmology Statement for Use of Animals in Ophthalmic and Vision Research. The rats were placed in individual plastic cages in a temperature-controlled room (22°C) in which a 12-12-h light-dark cycle was maintained. Appropriate food and water were provided to the rats.

### Alkali burn model

The procedures were performed under general anesthesia induced by intramuscular injection of ketamine hydrochloride (35 mg/kg) and xylazine hydrochloride (5 mg/kg). CNV was induced according to a previously described cauterization technique using silver nitrate^(8)^. The corneas of the rats were cauterized with a chemical applicator stick of 2-mm-diameter consisting of 75% silver nitrate and 25% potassium nitrate. This stick was touched onto the central corneas for 8 s under an operating microscope. After cauterization, the corneas and fornices were irrigated with 10 ml of normal saline to remove any residual silver nitrate. The rats were categorized randomly into three groups of 10 as follows: Group 1 (n=10) rats were treated topically with bevacizumab (Altuzan^®^ 400 mg/16 ml, F. Hoffmann-La Roche Ltd., Basel, Switzerland) solution at a concentration of 10 mg/ml (twice daily), Group 2 (n=10) rats were treated topically with CsA 0.1% eye drops (Depores X, Deva Inc) (twice daily), and Group 3 (control group, n=10) rats were treated with saline solution (0.9%) (twice daily) to both eyes. All procedures were performed by the same investigator (N.K.).

### Clinical and histological examination

All rats were subjected to slit-lamp examination on the 3rd and 28th day. On the 3rd day, using the method similar to that used by Manzano et al, the extent of burn stimulus response was graded for each cornea by slit-lamp examination as follows: grade 0 (no blister, not raised above the corneal surface), grade 1 (small blister, raised slightly above the surface), grade 2 (medium blister, raised moderately above the surface), and grade 3 (large blister)^(9)^.

On the 28th day of examination, corneal edema and corneal opacity grades were evaluated based on biomicroscopic examination using the method described by Yoeruek et al.^(10)^ Corneal opacity was graded for each cornea as follows: grade 0 (transparent), grade 1 (minimal haze, details of iris and pupil distinct), grade 2 (mild haze, iris and pupil detectable), grade 3 (moderate haze, iris and pupil hardly visible), and grade 4 (opaque, iris and pupil not discernable)^(10)^. Thereafter, the rats were sacrificed using a high dose of pentothal sodium (Pentothal^®^, Abbott, Italy). Eyes were removed and placed in 10% formaldehyde for 24 h. The corneas were then excised from the limbus, and 5-µm-thick corneal sections were prepared. The sections were sliced from both the central region of the burn area and the intensive neovascularization area. The thickness of the corneal layer was measured using the ImageJ program on the histological sections. They were then stained with hematoxylin-eosin and Masson’s trichrome^(11)^. The corneal quadrants were evaluated under 400× magnification, and the number of blood vessels, degree of inflammation, and collagen formation were compared^(11)^. Inflammation is accompanied by cellular chemotaxis, migration, and proliferation in a controlled manner through proinflammatory and anti-inflammatory molecules. Inflammation was graded for each cornea as follows: grade 0 (no inflammation), grade 1 (mild-to-moderate inflammation), and grade 2 (severe inflammation). Collagen formation was also graded for each cornea as follows: grade 0 (regular), grade 1 (minimal separation and disruption), and grade 2 (severe disruption).

### Statistical analysis

Statistical analysis was conducted using the Turcosa software (Turcosa Analytics, Turkey). Convenience of the data to normal distribution was evaluated using the Shapiro-Wilk test. Comparisons among the groups were performed by one-way analysis of variance, followed by *post hoc* Tukey’s multiple comparison test. A p-value <0.05 was considered to be statistically significant.

## RESULTS

**Figure 1:**
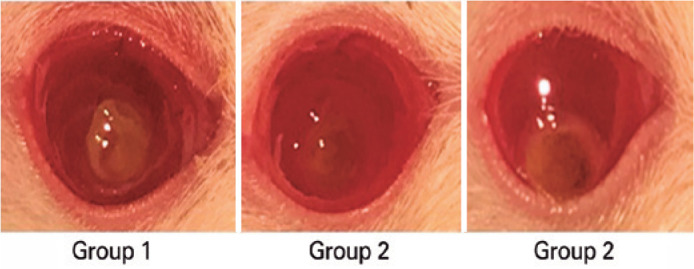
The degree of corneal neovascularization according to the groups on the 3rd day.

The degree of CNV is depicted in figure 1. All groups had corneal burn grades of 2 and 3.

Corneal opacity grade was statistically significantly lower in the CsA treatment group (Group 2) than in the control group (Group 3) (p=0.04) (Table 1). The corneal edema grade was also statistically significantly lower in the CsA treatment group than in the control group (p=0.00) (Table 1).

**Table 1:** Corneal opacity and edema grades according to study groups

	Group 1 (n=10)	Group 2 (n=10)	Group 3 (n=10)	p-value
Corneal opacity grades	1.70 ± 1.05^ab^	1.30 ± 0.94^b^	2.30 ± 0.48^a^	0.04
Corneal edema grades	0.60 ± 0.69^ab^	0.10 ± 0.31^b^	1.10 ± 0.31^a^	0.00

**Figure 2:**
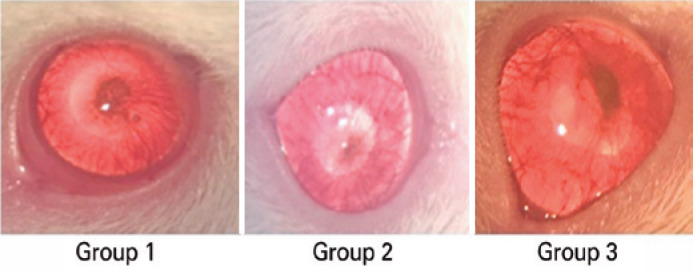
Image of an eye with corneal neovascularization after 28 days of treatment.

Histopathological examination revealed the average numbers of blood vessels as 1.55 ± 1.2 in Group 1, 1.34 ± 1.57 in Group 2, and 2.71 ± 2.7 in Group 3 (Figure 2). Group 2 had significantly fewer blood vessels than Group 3 (p=0.001) (Table 2). Regarding the evaluation of collagen formation by Masson’s trichrome staining, the CsA treatment group demonstrated more regular collagen formation than the bevacizumab treatment group and control group (p=0.03) (Table 3).

**Table 2:** The average number of blood vessels according to study groups

	Group 1 (n=10)	Group 2 (n=10)	Group 3 (n=10)	p-value
Vessels	1.00 (0.00-3.00)^ab^	1.00 (0.00-2.00)^b^	2.00 (0.75-4.00)^a^	0.001

Different superscripts in a row indicate statistically significant difference.

**Table 3:** Collagen formation evaluation by Masson’s trichrome staining

	Group 1 (n=10)	Group 2 (n=10)	Group 3 (n=10)	p-value
Collagen formation	1.10 ± 0.73^a^	0.50 ± 0.70^b^	1.60 ± 0.51^a^	0.03

Different superscripts in a row indicate statistically significant difference.

**Figure 3:**
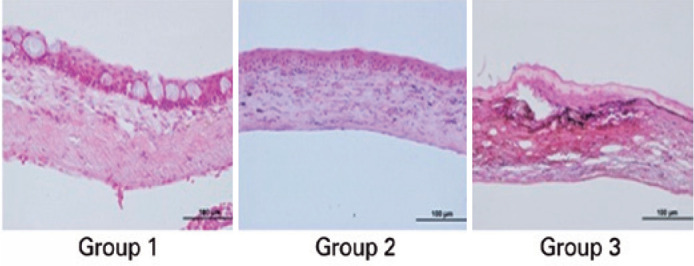
Histopathological evaluation of corneal inflammation.

The results of the histopathological evaluation of corneal inflammation are shown in figure 3 and table 4. The lowest corneal inflammation grade was identified in the CsA treatment group, followed by the bevacizumab treatment group and control group. The corneal inflammation grade was statistically lower in the CsA treatment group and the bevacizumab treatment group than in the control group (p=0.014 and 0.001, respectively).

**Table 4:** Corneal inflammation grades according to study groups

	Group 1 (n=10)	Group 2 (n=10)	Group 3 (n=10)	p-value
Corneal inflammation grades	0.90 ± 0.73^b^	0.40 ± 0.51^b^	1.7 ± 0.48^a^	0.014 0.001

Different superscripts in a row indicate statistically significant difference.

The thickness of the corneal layer was measured using the ImageJ program on the histological sections. The mean corneal thickness was 129.54 ± 32.35 in the bevacizumab treatment group, 110.76 ± 28.73 in the CsA treatment group, and 132.57 ± 33.52 in the control group. The thickness of the corneal layer was statistically lower in the CsA treatment group than in the bevacizumab treatment group and control group (p=0.001) (Table 5).

**Table 5:** The thickness of the corneal layer according to study groups

	Group 1 (n=10)	Group 2 (n=10)	Group 3 (n=10)	p-value
Corneal thickness	129.54 ± 32.35^a^	110.76 ± 28.73^b^	132.57 ± 33.52^a^	0.001

Different superscripts in a row indicate statistically significant difference.

## DISCUSSION

In this study, we designed an experimental model of CNV in rats and applied topical bevacizumab 1% and/ or topical CsA 0.1% to determine their effects on newly formed vessels and also compared their effects to those in the control group. To the best of our knowledge, this is the first study to use topical CsA 0.1% in rat eyes. Our findings demonstrated that topical CsA 0.1% was the most effective agent, and the efficacy of topical bevacizumab 1% was found to be superior to that of topical saline solution in reducing CNV and corneal inflammation.

CNV is a clinical condition that, when left untreated, leads to significant visual deficit^(12)^. To maintain corneal avascularity, angiogenic factors and inhibitors must maintain homeostasis. VEGF, fibroblast growth factors, CD3-positive cells (T lymphocytes), extracellular matrix metalloproteinases, cyclooxygenase 2, interleukin 2, and tumor necrosis factor are some of the endogenous activators in ocular angiogenesis. In addition, interferons, interleukin-12, endostatin, angiostatin, and thrombospondin play a role in the inhibition of ocular angiogenesis^(13)^. Amano et al. reported that the level of VEGF increased with trauma in rat corneas and neovascularization was associated with VEGF^(14)^. VEGF is a family of proteins comprising VEGF-A, -B, -C, and -D, the viral VEGF homolog VEGF-E, and the placental growth factor^(15)^. VEGF-A is one of the most important mediators of angiogenesis. Its expression is upregulated under conditions of neovascularization, and it plays a vital role in the development of pathological angiogenesis in inflammatory, neoplastic, and vascular diseases of the eye. In multiple animal studies and clinical trials, especially in cases unresponsive to conventional anti-inflammatory medications, several anti-VEGF agents such as bevacizumab have been used off-label for the treatment of CNV^(7,16)^. Bevacizumab is known to inhibits all isoforms of VEGF-A^(17)^. There are also data indicating the topical, subconjunctival, and intrastromal administration of bevacizumab at varying doses for the treatment of CNV^(18)^. Although no differences were reported between the topical and subconjunctival administration of bevacizumab^(10)^, Kim et al. reported in their study that topical administration of bevacizumab continues its activity for a much longer period than subconjunctival administration^(19)^. Topical administration is potentially safer than the subconjunctival injection that harbors a risk of severe adverse effects^(20)^. Moreover, in humans, topical agents can be self-administered. The results of our study were found to be consistent with the literature as anticipated. In our study, the histopathological examination revealed a statistically significant reduction in the intensity of inflammation, fibroblast activity, and number of blood vessels in the bevacizumab-treated groups compared with the control group.

CsA is a cyclic undecapeptide drug that inhibits the activity of transcription factors of the nuclear factor of the activated T-cell family, and it has long been used successfully as a systemic immunomodulator. Topical ophthalmic emulsion of CsA at a dose of 0.05% was appro ved by the Food and Drug Administration to treat dry eye disease in 2003. Different concentrations of topical CsA have also been examined for the treatment of various ocular surface inflammatory disorders, including atopic keratoconjunctivitis, acute corneal graft failure, and graft-versus-host disease^(21-23)^.

Graft rejection is the most common cause of corneal graft failure in the late postoperative period. Several corneal grafts in recipients with CNV undergo rejection^(24)^. CNV appears to be a trigger for corneal graft rejection. In addition, it has been shown that topical application of CsA prolongs corneal graft survival in an experimental study. Hernández et al. demonstrated that systemic CsA administration inhibits the migration of primary endothelial cells and angiogenesis induced by VEGF^(25)^. The authors speculated that this effect appears to be mediated by the inhibition of cyclooxygenase (Cox)-2, whose transcription is activated by VEGF in primary endothelial cells. Earlier, Benelli et al. showed that topical administration of CsA 4% inhibited CNV in a rat xenotransplantation model^(26)^. Lipman et al. evaluated the effect of CsA in an experimental CNV model stimulated with interleukin-2^(27)^. They reported that 25 mg/ kg CsA in olive oil noticeably reduced CNV compared to that in the control group. In a rat CNV model, Bucak et al. demonstrated that topical administration of CsA 0.05% was macroscopically and histologically effective in treating CNV^(28)^. However, the therapeutic potential of topical CsA 0.1% on CNV has not been explored till date. In our study, we observed that topical administration of CsA 0.1% was more effective in inhibiting CNV than the topical administration of bevacizumab 1%. The lower efficiency of topical bevacizumab than CsA in the present study may be because topically applied bevacizumab has limited capacity to penetrate the cornea with an intact epithelium, which may delimitate its antiangiogenic effects^(29)^. Topical bevacizumab has a molecular weight of 149 kDa, and its molecules are too large to penetrate the tight junctions of the intact corneal epithelium. In addition, it may be due to the rapid washout of bevacizumab drops from the corneal surface and the short-term contact of bevacizumab with the damaged cornea. However, it has been reported that bevacizumab may have undesirable effects, including suppression of wound healing and corneal nerve regeneration, and can systemically cause hypertension and cardiovascular disease^(30)^.

In conclusion, our study demonstrated that topical CsA 0.1% administration was macroscopically and histologically effective in treating CNV in rats. Hence, topical CsA 0.1% eye drops may play a role in the treatment of CNV in humans. However, further studies are required to provide additional evidence regarding the inhibitory effects of topical CsA on CNV.
